# The Oxford Knee Score is a reliable predictor of patients in a health state worse than death and awaiting total knee arthroplasty

**DOI:** 10.1186/s42836-022-00132-9

**Published:** 2022-08-03

**Authors:** N. D. Clement, I. Afzal, P. Liu, K. M. Phoon, V. Asopa, D. H. Sochart, D. F. Kader

**Affiliations:** 1 Edinburgh Orthopaedics, Royal Infirmary of Edinburgh, Little France, Edinburgh, EH16 4SA UK; 2 Southwest London Elective Orthopaedic Centre, Epsom, UK

**Keywords:** Oxford knee score, Arthroplasty, Knee, Outcome, Worse than death

## Abstract

**Background:**

The health-related quality of life of patients awaiting a total knee arthroplasty (TKA) deteriorates with increasing time to surgery and identification of those with the worst quality of life may help to prioritize patients. The aims were to identify and validate independent variable(s) associated with a health state worse than death (WTD) in patients awaiting a TKA and whether these variables influenced patients-reported outcome measures.

**Methods:**

A retrospective cohort of 5857 patients undergoing a primary TKA was identified from an established arthroplasty database. Patient demographics, body mass index (BMI), index of multiple deprivation, Oxford Knee Score (OKS), EuroQoL five dimension (EQ-5D) 3 level, and visual analogue scale (EQ-VAS) were collected preoperatively and one year postoperatively. An EQ-5D utility of less than zero was defined as WTD. A randomly selected subset of patients (*n* = 3076) was used to validate the variable that was most predictive of a state WTD and to assess the influence on patient-reported outcomes.

**Results:**

There were 771 (13.2%) patients with a health state WTD. Increasing social deprivation (*P* = 0.050), worse preoperative OKS (*P* < 0.001), or EQ-VAS (*P* < 0.001) were independently associated with a health state WTD. The OKS was the most reliable predictor (area under curve 88.9%, 95% CI 87.8 to 90.1, *P* < 0.001) of a health state WTD. A threshold value of 16 or less, 80% sensitive and specific, was validated and confirmed to have a negative predictive value of 97.5%. Patients with an OKS of 16 or less had a significantly greater improvement in their OKS (difference 6.9, *P* < 0.001) and EQ-5D score (difference 0.257, *P* < 0.001). When adjusting for confounding factors, a health status WTD was not associated with worse postoperative OKS (difference –0.6, 95% CI –1.4 to 0.3, *P* = 0.177), EQ-5D (difference –0.016, 95% CI –0.036 to 0.003, *P* = 0.097) or patient satisfaction (difference –1.8, 95% CI –4.3 to 0.7, *P* = 0.162).

**Conclusion:**

A threshold score 16 or less in OKS was a reliable predictor of a health status WTD and was associated with a greater improvement in knee-specific and health-related quality of life following TKA.

## Introduction

The coronavirus disease 2019 (COVID-19) pandemic has resulted in an unprecedented number of patients on waiting lists for elective surgery, due to the disruption to healthcare services [1]. Knee arthroplasty is a cost-effective intervention that improves physical function and delivers pain relief to patients with end-stage arthritis of the knee [2]. Failure to deliver timely arthroplasty surgery results in a diminished quality of life and therefore being on a waiting list is not a benign process [3]. The number of patients on the waiting list for a knee arthroplasty and living in a health state worse than death (WTD) has nearly doubled to 22% in the National Health Service in the UK during the COVID-19 pandemic [4]. This may be due to the prolonged negative consequences of living with chronic pain resulting in deterioration of physical and mental health [5]. Increasing use of opioid analgesia to manage escalating pain may also exert negative effects on a patient’s quality of life [5, 6]. For these reasons, continual assessment of patients on waiting lists is advised to identify patients failing to cope and to reprioritize their surgery according to their clinical need [7, 8].

The Federation of Surgical Specialty Association has defined surgical priority according to four levels [9]. Priority one is emergency care, which may not apply to planned arthroplasty surgery [9]. However, priority two surgery (within a month) applies to arthroplasty patients where delays may prejudice their outcome; priority three (less than three months) applies to avascular necrosis of the hip: and priority four (more than three months) applies to uncomplicated arthroplasty [9]. All patients listed for knee arthroplasty are not equal and priority four will not be applicable to all patients [10]. Objective prioritization of patients is difficult, and authors have suggested prioritizing according to scores, applied ethics, and knee-specific function [8, 11–13]. Prior studies have identified which patients may benefit from a total knee arthroplasty (TKA). However, these have been more inclusive than exclusive and do not aid in prioritizing patients [14–16]. The ability to identify patients who are failing to cope and living in a health state WTD and may thereby have maximal benefit from a TKA, would enable optimal use of limited resources.

The primary aim of this study was to identify independent variables associated with a health state WTD in patients awaiting a TKA. The secondary aims were to (1) identify and (2) validate the most reliable variable associated with a health state WTD, and (3) to assess whether this influenced the patient-reported outcomes following TKA.

## Methods

Patients for this study were identified retrospectively from a prospectively compiled arthroplasty database held at the study centre. During a 9-year period (June 2007 to November 2016), 5857 patients undergoing primary TKA at the study centre were asked to complete a preoperative patient questionnaire. There were 2204 male patients and 3653 female patients with a mean age of 71.1 (standard deviation [SD] 9.0, range, 30 to 96) years. The mean body mass index (BMI) was 30.6 (SD 5.5) kg/m^2^. Socioeconomic deprivation was measured using Index of Multiple Deprivation (IMD) deciles (1 being the most deprived and 10 being the least deprived) [17].

The Oxford knee score (OKS) [18] and EuroQoL (EQ) health related quality of life (HRQoL) questionnaire [19] were recorded preoperatively and at one year postoperatively. The OKS consists of twelve questions that are assessed using a Likert scale with values from 0 to 4, a summative score is then calculated where 48 is the best possible score (least symptomatic) and 0 is the worst possible score (most symptomatic) [20]. The minimal clinically important difference (MCID) is the smallest change of a score to be of importance to the patient, and has been defined for the OKS as a difference of 5 points [21]. The EuroQoL (EQ) general health questionnaire evaluates five dimensions (5D), which include: mobility, self-care, usual activities, pain/discomfort and anxiety/depression. The 3L version of the EuroQoL questionnaire was used, with the responses to the five dimensions being recorded at three levels of severity [19]. An individual patient’s health state can be reported based on a three-digit code for each domain, of which there are 243 possible health states [19]. Each health state was converted to a single summary index that was specific to the United Kingdom (UK) population and is based on a time trade-off technique. This index is on a scale of –0.594 to 1, where 1 represents perfect health, and 0 represents death. Negative values represent a state perceived as WTD [22]. The MCID in the EQ-5D is defined as 0.08 [23]. The EQ visual analogue scale (VAS) was also assessed preoperatively which rates the patients’ subjective health from 100 (best) to 0 (worst) imaginable health state.

Patients were randomly selected from the cohort to validate the identified threshold value in the OKS that was predictive of a health state WTD. Patients were assigned a study number and a random number generator was used (www.random.org) to identify the group according to the power calculation [24]. This group included 3076 patients of which 1194 (38.2%) were male and 1933 (61.8%) were female, with a mean age of 70.5 (SD, 9.0; range, 32 to 94) years. This group was also used to compare the patient-reported outcomes according to the threshold value.

### Statistical analysis

Statistical analysis was performed using Statistical Package for Social Sciences version 17.0 (SPSS Inc., Chicago, IL, USA). The data assessed demonstrated a normal distribution and parametric tests were used to assess continuous variables for significant differences between groups. A Student’s *t*-test, unpaired and paired, was used to compare linear variables between groups. Dichotomous variables were assessed using a Chi-square test. Pearson’s and Spearman’s correlations (ordinal data) were used to assess the relationships between scalar variables. Multivariate Logistic regression analysis was used to identify independent predictors of health state WTD. Multivariate linear regression analyses (MVLRA) were used to assess independent association of a health state WTD with patient-reported outcomes (OKS, EQ-5D and satisfaction). Variables were assessed for multicollinearity and the variance inflation factor was below 2 for all included factors. Receiver operating characteristic (ROC) curve analysis was used to identify thresholds in the OKS, EQ-VAS and IMD that identified a health state WTD. The area under the ROC curve (AUC) ranges from 0.5, indicating a test with no accuracy in distinguishing whether a patient was satisfied or if their expectation was fulfilled, to 1.0 where the test was perfectly accurate identifying all patients with a health state WTD. The threshold was defined as the point (OKS) at which the sensitivity and specificity were maximal in predicting a health state WTD. A *P*-value of < 0.05 was defined as statistically significant.

A power calculation for the validation cohort was performed using an effect size of 0.2, two-tailed analyses, and alpha of 0.05 (with Bonferroni correction for multiple testing), an allocation of 1:3 (due to lower proportion of patients having an OKS of less than 16) and a power of 95% would require a minimum of 2578 patients (645 *vs.* 1933).

There was no additional patient contact and as such this project was performed as a service evaluation without the need for formal ethical approval. The project was registered with the institutions audit department and was conducted in accordance with the Declaration of Helsinki and the guidelines for good clinical practice.

## Results

**Fig. 1:**
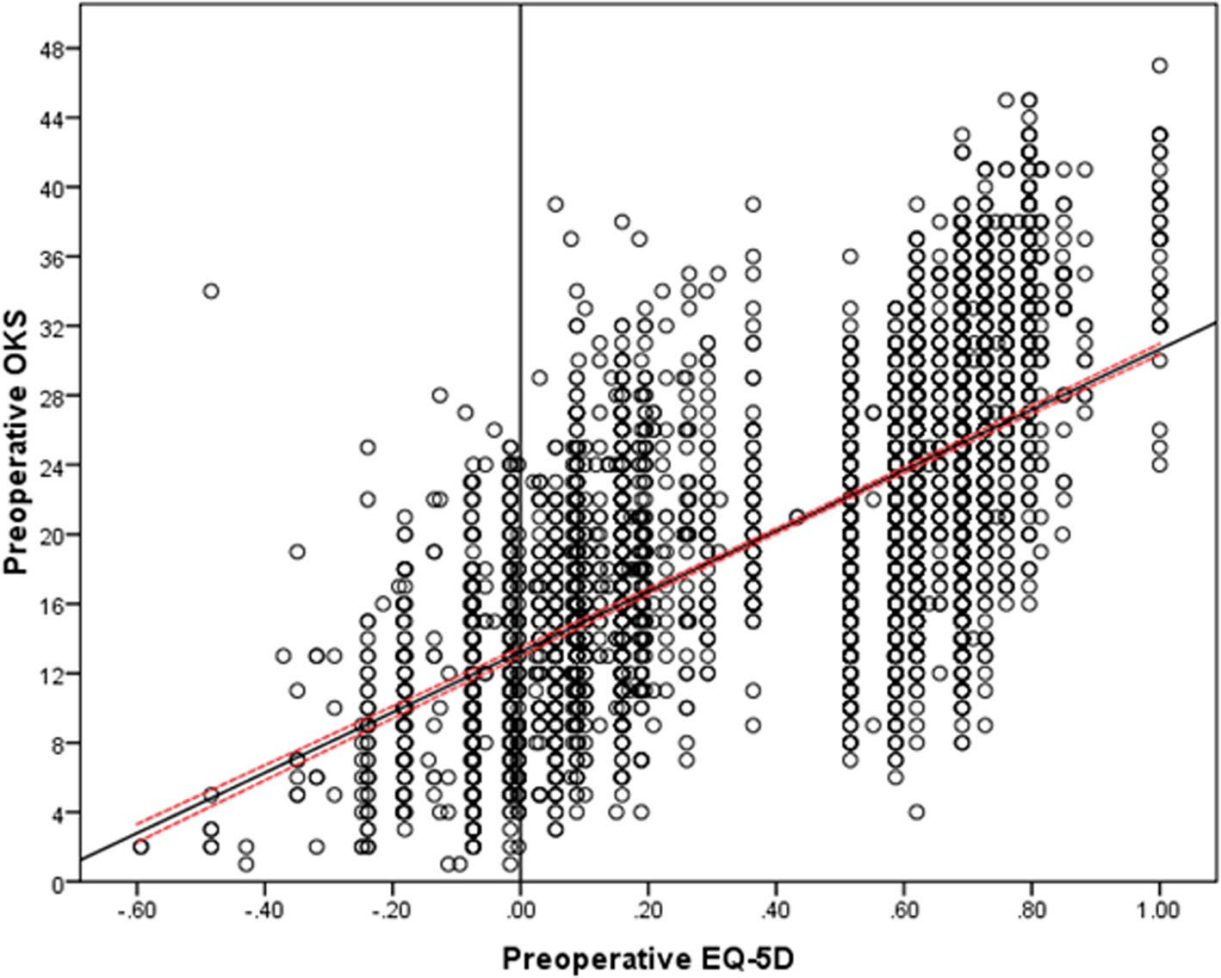
Scatter plot with linear line of the best fit (black line) and 95% confidence intervals around the mean (red dashed lines) for preoperative EQ-5D and the OKS

**Fig. 2:**
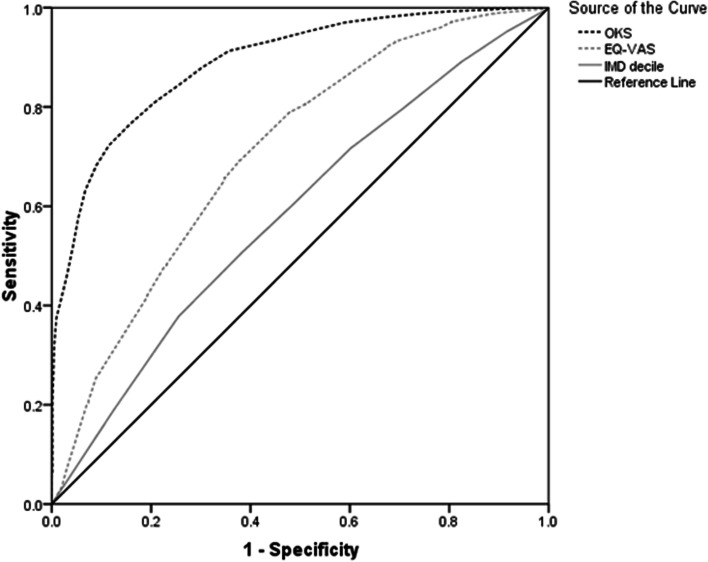
Receiver operating characteristic curves for OKS, EQ-VAS and indices of multiple deprivation as predictors of a health state WTD

**Fig. 3:**
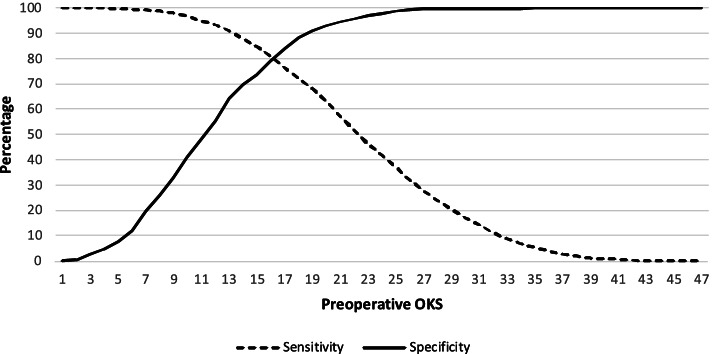
Sensitivity and specificity plot for the preoperative OKS as a predictor of a health state WTD. Red line represents the threshold value on the OKS that offers the greatest sensitivity and specificity

There were 771 (13.2%) patients with a preoperative HRQoL state WTD. Unadjusted analysis demonstrated that female gender (*P* < 0.001), younger age (*P* < 0.001), increasing BMI category (*P* < 0.001), increasing social deprivation (*P* < 0.001), and worse preoperative OKS (*P* < 0.001), EQ-5D (*P* < 0.001) or EQ-VAS (*P* < 0.001) were associated with a state WTD (Table 1). However, when adjusting for confounding factors, only increasing social deprivation (*P* = 0.050), worse preoperative OKS (*P* < 0.001), or EQ-VAS (*P* < 0.001) were independently associated with a health state WTD (Table 2). There were significant (*P* < 0.001) correlations with preoperative EQ-5D and the OKS (*r* = 0.68, Pearson), EQ-VAS (*r* = 0.32, Pearson) and indices of multiple deprivation (*r* = 0.15, Spearman), with the greatest correlation being observed with the OKS (Fig. 1). The OKS was the most reliable predictor of a health status WTD in patients awaiting a TKA, with an AUC of 88.9%, when compared to the EQ-VAS and indices of multiple deprivation (Table 3) (Fig. 2). The point of maximal sensitivity and specificity in the preoperative OKS was 16 or less, which was equivalent to 80% sensitivity and specificity (Fig. 3).

**Table 1:** Unadjusted analysis assessing preoperative variables associated with a preoperative health status of worse than death in patients awaiting a total knee arthroplasty

Variable			Worse than Death		Difference/Odds Ratio (95%CI)	*P*-value
			No (*n*=5086)	Yes (*n*=771)		
**Gender** (*n*, % of group)		Male	2003 (39.4)	201 (26.1)	1.84 (1.55 to 2.19)	<0.001*
		Female	3083 (60.6)	570 (73.9)		
**Age** (years: mean, SD)			71.3 (8.9)	70.0 (9.9)	1.2 (0.6 to 1.9)	<0.001**
**BMI **(n, % of group)	Underweight		19 (0.4)	5 (0.6)		<0.001*
	Normal		632 (12.4)	111 (14.4)		
	Overweight		1876 (36.9)	232 (30.1)		
	Obese I		1665 (32.7)	228 (29.6)		
	Obese II		604 (11.9)	122 (15.8)		
	Obese III		290 (5.7)	73 (9.5)		
**Indices of multiple deprivation decile** (*n*, % of group)		1 (most)	25 (0.5)	5 (0.6)		<0.001*
		2	216 (4.2)	59 (7.7)		
		3	312 (6.1)	71 (9.2)		
		4	485 (9.5)	92 (11.9)		
		5	401 (7.9)	80 (10.4)		
		6	586 (11.5)	91 (11.8)		
		7	471 (9.3)	76 (9.9)		
		8	666 (13.1)	100 (13)		
		9	952 (18.7)	100 (13)		
		10 (least)	972 (19.1)	97 (12.6)		
**Preoperative**		**OKS**	22.3 (7.3)	11.2 (5.2)	11.0 (10.5 to 11.6)	<0.001**
(mean, SD)		**EQ-5D**	0.511 (0.255)	-0.075 (0.093)	0.586 (0.568 to 0.605)	<0.001**
		**EQ-VAS**	70.8 (17.4)	55.2 (21.8)	15.6 (14.2 to 17.0)	<0.001**

*BMI* Body mass index, *CI* Confidence intervals, *EQ-5D* EuroQol 5-dimension, *EQ-VAS* EuroQol visual analogue scale, *OKS* Oxford Knee Score, *SD* standard deviation

^*^ chi square test

^**^ unpaired *t*-test

**Table 2:** Logistic regression analysis was used to assess the independence of preoperative variables associated with a health state WTD in patients awaiting a total knee arthroplasty. All preoperative variables from Table 1 were entered into the model (Nagelkerke *R* ^2^ = 0.46)

**Variable**		**OR**	**95% CI**		***P*** **-value**
			**Lower**	**Upper**	
**Gender**	Male	Reference			
	Female	0.87	0.71	1.08	0.205
**Mean Age **		0.99	0.98	1.00	0.221
**BMI**		0.98	0.97	1.00	0.133
**IMD decile**		0.96	0.93	1.00	0.050
**Preoperative**	**OKS**	0.77	0.75	0.78	<0.001
	**EQ-VAS**	0.98	0.97	0.98	<0.001

*BMI* Body mass index, *CI* Confidence intervals, *EQ-VAS* EuroQol visual analogue scale, *IMD* Indices of multiple deprivation decile, *OKS* Oxford Knee Score, *OR* Odds ratio

**Table 3:** Receiver operating characteristic curve analysis for predicting a health status WTD in patients awaiting a total knee arthroplasty according to their OKS, EQ-VAS and index of multiple deprivation decile

Variable	AUC	95% CI		*P*-value
		**Lower**	**Upper**	
**Preoperative OKS**	88.9	87.8	90.1	< 0.001
**Preoperative EQ-VAS**	70.7	68.6	72.8	< 0.001
**IMD decile**	58.4	56.3	60.6	< 0.001

*AUC* Area under the curve, *CI* Confidence intervals, *EQ-VAS* EuroQol visual analogue scale, *IMD* Indices of multiple deprivation decile, *OKS* Oxford Knee Score

In the validation cohort, there were 370 (12.0%) patients with a preoperative HRQoL state WTD, which was not significantly different from the overall cohort (*P* = 0.127, Chi square), and 957 (31.1%) had a preoperative OKS of 16 or less. The threshold value of 16 or less in the OKS was 85.7% specific and 76.3% sensitive in predicting a health state WTD and had a negative predictive value of 97.5%. Patients with a preoperative OKS of 16 or less had a significantly greater improvement in their OKS and EQ-5D score postoperatively when compared to those who had a higher score which was beyond the MCID (Table 4). Despite the greater improvement in these scores, those with a preoperative OKS of 16 or less had a significantly worse postoperative OKS and EQ-5D score (Table 4), which were due to their significantly worse preoperative scores. Furthermore, those patients with a preoperative OKS of 16 or less had a significantly worse (lower rating of) satisfaction with the outcome of their operation (Table 4). However, when adjusting for confounding factors (Table 1) a health status WTD was not associated with worse postoperative OKS (difference, –0.6; 95% CI –1.4 to 0.3; *P* = 0.177, MVLRA), EQ-5D (difference, –0.016, 95% CI, –0.036 to 0.003; *P* = 0.097, MVLRA) or patient satisfaction (difference, –1.8; 95% CI, –4.3 to 0.7; *P* = 0.162, MVLRA). No patient had a health state WTD postoperatively.

**Table 4:** Patient-reported outcome measures according to the preoperative threshold value on the OKS

**Functional Measure**	**OKS** (mean, SD)		**Difference** (95% CI)	***P*** -value*
	** > 16** (*n* = 2119)	**≤** **16** (*n* = 957)		
**Preoperative OKS**	24.8 (5.7)	11.7 (3.4)	13.1 (12.7 to 13.5)	< 0.001
**Postoperative OKS**	38.4 (8.0)	32.2 (10.5)	6.2 (5.5 to 6.9)	< 0.001
**Difference**	13.6 (8.4)	20.5 (10.5)	6.9 (6.2 to 7.6)	< 0.001
***P*** -value**	< 0.001	< 0.001		
**Preoperative EQ-5D**	0.575 (0.231)	0.169 (0.268)	0.406 (0.387 to 0.424)	< 0.001
**Postoperative EQ-5D**	0.818 (0.204)	0.667 (0.286)	0.150 (0.133 to 0.168)	< 0.001
**Difference**	0.242 (0.277)	0.499 (0.340)	0.257 (0.234 to 0.280)	< 0.001
***P*** -value**	< 0.001	< 0.001		
**Satisfaction**	87.8 (16.9)	83.7 (19.5)	4.2 (2.8 to 5.6)	< 0.001

*CI* Confidence intervals, *EQ-5D* EuroQol 5-dimension, *OKS* Oxford Knee Score, *SD* standard deviation

^*^ unpaired *t*-test

^**^ paired *t*-test

## Discussion

This study has demonstrated that 13.2% of patients waiting for a TKA, prior to the COVID-19 pandemic, were in a self-defined health state WTD, which was associated with increasing social deprivation and worse knee-specific symptoms (OKS). The OKS was a reliable predictor of a health status WTD and a score of 16 or less offered 80% sensitivity and specificity, which was validated and shown to have a negative predictive value of 97.5%. Patients with a preoperative OKS of 16 or less had a greater improvement in their OKS and EQ-5D scores, and although they had worse postoperative scores and a lower level of satisfaction with the outcome of their operation, when adjusting for confounding factors these outcomes were equal to those with an OKS of more than 16 points.

Rosser and Kind [25] were amongst the first to recognize the importance of health state WTD in the context of limited resources and health economic assessments. The EQ-5D UK value set is based on hypothetical valuations of health states according to the general public’s opinion [19]. The current UK EQ-5D-3L value set was developed based on data collected in the early 1990s and has values that range from 1 (for no problems) to − 0.594 (for the worst health state) for all five dimensions assessed [19]. However, it is recognized that approximately one third of health states assigned to the 243 possible health states are negative values, and therefore represent a health state WTD [26]. This percentage is greater than that observed in other countries, using their own culture-specific time trade-off utility scores, but may reflect genuine preferences of the public assessed [26]. Nonetheless, for the purposes of the current study those with the worst HRQoL were reliably identified using the OKS and whether those patients were in a state WTD may be debatable.

The percentage of patients in a health state WTD observed in the current study (13%) is supported by Scott *et al*. (2019) [22], who described a percentage of 12% in their cohort of 2168 patients. Both of these percentages represent patients on pre-COVID-19 waiting lists whereas more recently Clement *et al*. (2021) [4] demonstrated an increase to 22% following the first wave of the pandemic. The current study affirms the findings of Scott *et al*. (2019) [22] who also identified female sex, increasing BMI, increasing social deprivation, worse OKS and EQ-VAS to be associated with a health state WTD, and that the OKS was independently associated with a health state WTD. Scott *et al*. (2019) [22] also found a similar threshold value of 16.5 points in the OKS to be predictive of a state WTD, but with a marginally lower sensitivity (71% *vs.* 80%) and specificity (64% *vs*. 80%) than the current study. Furthermore, the current study validated this threshold using a smaller cohort of patients and demonstrated a similar sensitivity and specificity of 85.7% and 76.3% respectively, suggesting that the OKS is a reliable predictor of a health state WTD.

To use a patient-reported outcome measure such as the OKS to prioritize patients for TKA may not be an ideal tool and may be liable to patients “gaming” the system. The proposed threshold value would enable those with the worst quality of life to be identified and who would benefit the most from TKA. An OKS of 32 or more has been shown to be a reliable predictor of patients not requiring a TKA [27]. However, Eibich *et al*. (2018) [15] have demonstrated HRQoL gains with a preoperative OKS of 44 or less, but whether all gains are clinically meaningful is not clear [23]. Price *et al*. (2020) [16] more recently demonstrated that a preoperative OKS threshold value of 41 or less identified as many patients as possible who would have clinically meaningful improvement in their knee symptoms. Therefore, the OKS seems to be more inclusive than exclusive when identifying those who may benefit from a TKA. Not all patients on a waiting list for a TKA are, however, equal and prioritizing according to pain severity and effects on socioeconomic consequences is difficult [8, 10, 28]. Rationing of TKA is inevitable when there are limited resources and demand exceeds capacity [12]. Using the threshold of 16 or less in the OKS may help surgeons prioritize these patients for TKA and enable the most cost-effective use of limited resources [14].

Prioritizing patients on waiting lists has been recognized by several authors either by assigning surgical priority according to indications or using tools to help quantify prioritization [8, 11–13]. Such tools often encompass patient factors, time spent on waiting list, and surgical factors. Patient factors such as the quality of life should also be taken into account, which is supported by patient opinions [29], and the threshold value of 16 points or less in the OKS may be a useful tool to quantify this. For each 6-month period spent on the waiting list, a clinically significant deterioration in the patient’s quality of life occurs [4], and although these patients had an improved quality of life following arthroplasty, it may not have been to the same level as they would have achieved prior to their deterioration [22]. Therefore, justifying the need to prioritize patients waiting < 6 months, > 6 months and > 12 months as different categories is important. The health-related quality of life of patients currently on waiting lists across the UK is, according to the EQ-5D utility index, 0.24 for total hip arthroplasty and 0.34 for TKA (On the scale, one is perfect health and zero means death). This is a lower quality of life than those observed in other morbidities such as diabetes (0.78) [30], heart failure (0.64) [31], chronic obstructive pulmonary disease (0.52) [32] or stroke (0.40) [33]. However, unlike most of these morbidities, the effect of arthritis on a patient’s quality of life is reversible with surgery [2]. This was observed in the current study with significant improvement in quality of life and no patient was in a state WTD following surgery.

A recent study by Kulkarni *et al*. (2021) [34] highlighted the plight of those on NHS waiting lists, describing their experiences. They found that patients felt their physical and mental health had deteriorated, and that they had struggled to access doctors and obtain help with their pain management. Farrow *et al*. (2021) [6] highlighted the increased use of opioid analgesia in patients awaiting arthroplasty during the COVID-19 pandemic and potential negative ramifications of this following surgery. The implications of deteriorating quality of life, increasing use of opioid analgesia and deconditioning of patients on perioperative complications and functional outcomes are not clear. Length of hospital stay has been shown to have significantly increased by half a day when the wait for surgery increased by 59 days following the initial wave of COVID-19 [35]. If this trend was observed for patients who have waited longer than an extra 59 days this may have an impact on capacity and delivery of services within the current footprint.

There are several limitations to the current study. All outcomes pre- and postoperatively were prior to the COVID-19 pandemic and may not be fully reflective of patients currently awaiting surgery. The study did not assess the influence of patient comorbidities or American Society of Anesthesiologists grade on health-related quality of life, which may have influenced their quality of life pre- and/or postoperatively. A further limitation was that this study only assessed one year postoperative outcome scores which may not be reflected with longer term follow-up. However, this does seem to be the optimal time-point to assess patient-reported outcomes [36]. Finally, the retrospective design of the study is also a limitation, as it would have been beneficial to further assess those patients in health state WTD to see if this really was the case, including an evaluation of their mental well-being at the time.

## Conclusion

A threshold score 16 or less in OKS was a reliable predictor of a health status WTD and was associated with a greater improvement in knee-specific indicators and HRQoL following TKA. The OKS could be used as a tool to prioritize patients awaiting a TKA, identifying those with the worst quality of life.

## Data Availability

Data are available on request to the corresponding authors should it be required, but this will have to be authorized by the study centre audit team.

## Abbreviations

AUC: Area under the curve
BMI: Body mass index
CI: Confidence intervals
COVID-19: Coronavirus disease 2019
EQ-5D: EuroQol 5-dimension
EQ-VAS: EuroQol visual analogue scale
HRQoL: Health related quality of life
IMD: Indices of multiple deprivation decile
MCID: Minimal clinically important difference
MVLRA: Multivariate linear regression analyses
OKS: Oxford Knee Score
OR: Odds ratio
ROC: Receiver operating characteristic
SD: Standard deviation
TKA: Total knee arthroplasty
UK: United Kingdom
WTD: Worse than death
